# Freedom under algorithms: how unpredictable and asocial management erodes free choice

**DOI:** 10.3389/frai.2025.1582085

**Published:** 2025-08-05

**Authors:** Robert Donoghue

**Affiliations:** School of Business, Faculty of Social Sciences, University of Essex, Colchester, Essex, United Kingdom

**Keywords:** algorithmic management, artificial intelligence, resilience, freedom, work, gig economy, platform labour

## Abstract

This article examines the impact of algorithmic management on individual freedom. To orient this exploration, I draw on the (feminist) conception of liberty as the choosing subject. The central suggestion is that algorithmic management poses a serious threat to an indispensable part of the freely choosing subject: namely, it degrades the ability of subordinates to reasonably foresee the consequences of their choices and consequently, fully realise their personality. I call this phenomenon the ‘foresight endangerment problem’ and argue that it has both a technical and a social face. The technical face highlights the inherent unpredictability of advanced algorithms, including those that execute managerial functions. This issue is further complicated by the fact that as algorithms become more resilient and useful, their outputs grow increasingly opaque and unpredictable—what some refer to as the resilience-predictability paradox. The technical face is made manifest in the reported experiences of workers in the gig economy who describe experiencing unpredictable managerial decisions that they cannot anticipate nor easily contest. Subjection to such managerial randomness erodes their ability to make informed choices in service of their personal goals. The social face emphasises the consequences of disembedding managerial power from social relationships between humans to asocial relationships between humans and software. Subordinates of human managers enjoy a vast number of tools to predict managerial thinking that arise from the intricate and complex processes of social interaction. The disembedding process forecloses the use of these tools and fundamentally undermines the capacity of subordinates to promote their ends through free choice.

## 1 Introduction

There is no shortage of vocalised concern about the widespread invasion of algorithms (and other kinds of artificial intelligence technologies) into the many facets of our lives. Algorithms have indeed become a critical part of our social infrastructure (Burrell and Fourcade, 2021). They are used to administer welfare and social services, determine prison sentences, structure our social media engagements, detect banking fraud, and direct police deployments, among numerous other functions (Wenzelburger et al., 2022). It seems that nearly every new application of algorithms has been met with scepticism and genuine apprehension by researchers, policymakers, NGOs, and civil society itself—despite the revelatory claims about their potential by technological optimists (Bourne, 2020). The use of algorithms to manage subordinates, in settings like the workplace, is no exception. As algorithms continue to spread quickly across workplaces, global discontent with algorithmic management (sometimes shortened to ‘AM’) is growing rapidly (Grohmann et al., 2022).

Much of the conversation about AM has centred on the injustices it perpetuates—and rightly so. In the liberal society, technological advancement precedes contemplation about whether it should be adopted—therefore, critical reflection is always *ex-post* (Deneen, 2019). New technologies simply emerge on the scene, and it is only after their immersion do we investigate their bio-psycho-socio-economic implications. So far, the discussion around AM has largely focused on how this technology perpetuates the social harms of bias, discrimination, non-transparency, and non-accountability (Tsamados et al., 2021). What remains under-theorised is the impact of AM on the freedom(s) of subordinates (data subjects, workers, consumers, benefits recipients, etc.). In other words, does replacing human management with algorithmic management create a meaningful difference in freedom for those dependent on its decisions? If so, is this impact positive or negative?

There are potentially many reasons one might be concerned about the impact of algorithms on the freedom of subordinates. The tendency of algorithms towards bias may unequally distribute rewards and sanctions across social groups, thereby unjustly foreclosing opportunities to some based on immutable characteristics. Or algorithms may operate as powerholders with greater impunity in their decisions because they cannot as easily be held accountable—and subjection to a power that can act arbitrarily is considered by many to be the antithesis of freedom (Pettit, 2014; Hayek and Hamowy, 2020). This article narrows its focus to only one particularly concerning dynamic: namely, how algorithmic systems threaten the choice freedom of the subordinates they oversee.

This central objective herein, then, is to draw out some of the unique and serious threats posed by AM to the choice freedom of subordinates—and this is, in part, achieved through comparison with traditional human management. My core contention is that subordinates managed by algorithms suffer a degraded capacity to foresee the consequences of their choices, and foresight into the consequences of one’s choices is an indispensable component of individual freedom. I will refer to this phenomenon as the ‘foresight endangerment problem’. This problem, as I am formulating it here, is composed of two overarching claims which require further evidencing.

The first claim is that ‘foresight’ into consequences of one’s choices is indeed a constitutive and indispensable part of individual freedom. I locate this relationship between choice foresight and liberty in the ‘feminist’ conception of freedom as the choosing subject (Rosenfeld, 2025). To enjoy the genuine ability to project oneself into the world and act with autonomy, one must be able to make choices in service of his or her goals, dreams, beliefs, etc. Yet, this is only possible with a reasonable understanding of how to navigate to those ends through choices, which ultimately requires reasonable foresight into where our choices will take us.

The second claim is that subjection to the power of an algorithm endangers a subject’s choice-related foresight. This endangerment, I argue, has both a technical and social face. The technical face stems from the unpredictability of AI system outputs, a phenomenon that has received much critical attention. AITs, most notably in the form of algorithms, often become ‘black boxes’ where it is (virtually) impossible to for humans to understand how a decision was made. This dynamic is not only problematic in and of itself but is exaggerated by the ‘resilience-unpredictability’ paradox. This paradox refers to a serious trade-off that arises with the use of algorithms: to make them more resilient, and therefore more useful and functional in the real dynamic world, their outputs will inevitably become more unpredictable.

The second, social face of this problem has received less attention. I maintain, however, that there is great value in considering how subjection to an algorithmic decision-maker (epistemically) differs from a human one. Further scrutiny reveals a fundamental and highly significant discrepancy between these two kinds of (power) arrangements: the algorithm-subordinate relationship is an *asocial* relationship between a non-social and social agent, whereas the human-subordinate relationship is a *social* relationship between two social agents. The offloading of managerial duties from humans to algorithms, then, has a disembedding effect for those managed by the latter. Disembedded managerial power, I will attempt to show, poses serious negative epistemic consequences for the subordinated and ultimately endangers their choice foresight and individual freedom.

To fully visualise the technical and social faces of the foresight endangerment problem, I explore them throughout the article within the context of the workplace. The workplace is an exemplary setting for such reflection because domination and the loss of freedom are always acutely at risk in employment settings. Furthermore, the world of work is one domain where algorithmic management has been operative for a significant period of time and is rapidly expanding. Increasing numbers of workers find themselves under the prerogative of algorithms and automated decision-makers.

The article structure is as follows. Section II briefly reviews the choosing subject theory of freedom. Importantly, the ‘foresight condition’ is established as a necessary component of free choice which sets the table for the ‘foresight endangerment problem’. Section III introduces what I refer to as the technical face of the foresight endangerment problem. Here, I explore the technical aspects that render algorithmic outputs unpredictable, and why this is worsened by attempts to improve algorithmic resilience. Section IV explores the social face of the foresight endangerment problem. This face underscores how the disembedding of managerial power deprives subordinates of several tools that ehance their choice foresight. These tools directly emerge from the process of social interaction and are lost when a human manager is replaced by an algorithm.

## 2 The choosing subject theory of freedom

The first step in demonstrating that algorithmic management endangers individual freedom is to clarify what is meant by ‘freedom’. It is unlikely that this step could be taken without invoking controversy as the meaning of this word is highly contested. The history of philosophy contains numerous articulations that we could deploy as the entry point for our analysis (Wolff and Resnick, 2012, p. 151). Berlin (2014) suggested, in his oft-cited lecture *Two Concepts of Liberty*, that historians have tracked “more than two hundred senses of this protean word”—although he was remarkably able to reduce them down to two. It is obviously impossible to evaluate the threat of algorithmic management with every conception of freedom. Nor would it be appropriate to proclaim that algorithmic management endangers freedom *simpliciter*. I, therefore, take the third option of presenting how algorithmic management undermines individual freedom understood in terms of one specific conception, namely the notion of freedom as the choosing subject.

The decision to employ the choosing subject theory of freedom does not suggest that future research should rule out similar inquiries based on alternative conceptions. I make no argument that one theory of freedom has superior analytical value with respect to this topic. On the contrary, there is substantial value in exploring algorithmic management from several different theories of freedom. Indeed, the literature already offers elucidatory analyses of the unfreedoms inherent to algorithmically-managed digital platform labour from other philosophical traditions (Muldoon and Raekstad, 2022). It is merely my supposition that the choosing subject conception helpfully isolates and elucidates a particular threat posed by AM. Therefore, to fully capture that threat, the choosing subject lens is appropriate and necessary.

I will now briefly review the core premises of the choosing subject theory of freedom. Whilst its presentation will be inevitably truncated, we are able to obtain what we need from identifying three of its most basic premises. A review of those premises will reveal a key assumption that underpins this conception of freedom: something that I will refer to as the foresight condition. Establishing the foresight condition is an integral part of my argument because it is this condition of the free person which AM seems to undermine.

### 2.1 The doctrine of freedom as the choosing subject

The first premise underlying the choosing subject theory of freedom is a particular understanding of the individual: namely, that a ‘perspectival identity’ is a foundational aspect of the ‘autonomous person’. Friedman (2003) explains that this identity is comprised of one’s “perspective, outlook, or viewpoint, that is, [their] deeper, wants, desires, cares, concerns, values, and commitments” (Cathcart, 2023). In short, the summation of our ‘propositional attitudes’—i.e., our beliefs, desires, wishes, etc.—critically make up who we are as individuals (Oppy, 1998). Our perspectival identity iteratively conditions how we perceive, interpret, and judge the world around us, and it thereby plays a critical role in the formation of our personalities. Perhaps the notion of a perspectival identity could be likened to Martin Heidegger’s ‘fore-structure of understanding’ or Hans-Georg Gadamer’s concept of prejudice in that it is integral to shaping the subjectivity that governs our encounters with the external world (Weberman, 2000).

The link between a perspectival identity and the autonomous individual is straightforward: it would not make sense to describe an entity as (having the potential to be) autonomous if it lacked a perspectival identity. Consider, for instance, whether it would be appropriate to think of an iPhone as an autonomous agent. Whilst an iPhone is capable of information processing, it does not appear that the nature of this processing is generative of or associated with genuine propositional attitudes. The absence of such attitudes means that there is no possibility for an iPhone to act autonomously as there is no possibility for it to act in accordance with its self-derived preferences, beliefs, and other propositional attitudes. In other words, if autonomy is the capacity to ‘live one’s life according to reasons and motives that are taken to be one’s own’, then an entity capable of having reasons and motives (or propositional attitudes) is a presupposed logical necessity (Christman, 2020). Thus, a perspectival identity is foundational to the autonomous, free subject.

The second premise of the fully choosing subject theory of freedom is that we express or realise this core aspect of our being, our perspectival identity, through making choices.^1^ Indeed, it is hard to imagine how a perspectival identity could be expressed without the exercise of choice. As evidence of this assertion, consider why for the preponderance of human intellectual history freedom was defined in antithesis to the plight of the slave (Hayek and Hamowy, 2020). The slave identified in Roman jurisprudence as the archetype of unfreedom because her life choices are entirely alienated from any external arbitrary power in the Master (Skinner, 2019). Her life is not self-authored by her own choices but other-authored by the choices to whom she is totally subjugated. This means that every aspect of her daily life is completely dependent on the Master’s private whims as opposed to her autonomous propositional attitudes. The Master–Slave social arrangement constitutes an annihilation of the slave’s personality because the avenue for its expression is blocked: her perspectival identity (or will) cannot surface in the world through reasoned actions (or choices).

The third premise of this choosing subject theory is that for individuals to be free persons they must have sufficient social freedom to freely exercise their choices. The expression ‘social freedom’ here serves an important purpose: it circumscribes the contours of the choosing subject theory within the sprawling range of debates around individual liberty by identifying ‘social obstacles’ (as opposed to natural or intrapersonal obstacles) as the kind of constraint that is freedom-threatening. Pettit (2006) elaborates on this important aspect of theorising about freedom:

Suppose we want to know how much freedom someone enjoys in making a particular choice… we may be concerned with how far the agent’s access to the options given is unhindered on any front, whether in virtue of psychological pathology, physical incapacity, natural impediment, social constraint, or whatever.

As Pettit indicates, to ask when a person is free necessitates identifying those things that make them unfree. The choosing subject theory of freedom is fundamentally a theory of *social freedom*. That is, it identifies and advocates for the elimination of social obstacles that limit the free choice of individuals. Feminist philosopher Welch (2012), in developing the choosing subject theory, explicitly frames it in these terms:

…the importance of social freedom to individuals is the ability to shape their life and express their personality. Social freedom is essential for individuals to meaningfully live their lives by making plans, commitments, curious endeavours and inquiries, personal expressions, and relationships. These activities require that individuals be able to choose freely.

By focusing on social obstacles, the choosing subject conceptions stands beside other theories of social freedom like libertarian self-ownership, republican non-domination, anarchist mutualism, objectivist non-aggression, and so on. All these conceptions hold that it is only social obstacles that *compromise* the freedom of individuals, whereas other curtailments like a physical or psychological incapacities or natural impossibilities only *condition* it.

### 2.2 The foresight condition

In order for the argument of this paper to proceed, it is first necessary to highlight an important assumption underpinning the choosing subject theory of freedom. In so doing, we can more clearly see how subjection to the power of an algorithm endangers the freedom of subordinates. At root, the choosing subject theory presupposes that (for the free person) choices are made in a sequentially causal and intelligible environment. By emphasising choice as the mechanism for autonomously realising one’s personality, choices themselves must function as conceptualised in the theory. This would obviously include that choosers experience a causal and intelligible relationship between choices and outcomes. Put another way, the context in which one is embedded must permit persons to *navigate* to their preferred ends through their choices.

To illustrate the point, consider a thought experiment of *P* who is interested in improving her health. *P* decides to order the healthiest option on the menu when eating at a restaurant. However, *P* continually finds that what she is served is never what she ordered. Indeed, it appears to her as though what she receives is entirely random and completely unrelated to what she chooses and requests. (Let us also assume for the sake of simplicity that *P* must eat whatever is served—maybe she has a moral crusade against waste). This contrived and trivial scenario underscores a basic, but important, fact: choices are only a mechanism for the realisation of individual personality if they are exercised in a sequentially causal context that can be reasonably apprehended by the chooser. *P* can reasonably expect that when she chooses some item on the menu, it will ultimately be what she ends up eating. In this bizarre world, however, *P* is unable to manifest her desire and preference to eat healthy because her choices at T_1_ do not result in her chosen conditions at T_2_.

I suggest, then, that reasonable foresight is a necessary requisite of the free choosing subject. I formulate this necessity and assumption of the choosing subject theory as follows: the ‘foresight condition’ holds that individuals must be able to reasonably foresee the consequences of his or her choices for those choices to be considered freely made. It is necessary because absent the capacity, choices cannot be made in the service of one’s beliefs, dreams, hopes, and other propositional attitudes. Put another way, one’s choices cannot be made to advance a self-authored ‘rational life plan’—something widely regarded as essential to the moral goods of personhood and individuality (Francis and Francis, 1976; Wolff, 1991). A social context characterised by ‘randomness’ or ‘unpredictability’ jeopardises the capacity for individuals to enjoy the freedom of the choosing subject—something we will see algorithmic management is guilty of inducing.

The suggestion of this condition naturally begets a bright line problem: what is the threshold for ‘reasonable foresight’? How can we confidently know when an individual has sufficient foresight into the consequences of their choices to be deemed a fully free choosing subject? The world is clearly a dynamic place infused with randomness, leading to all kinds of possibilities that people could not have reasonably foreseen. Whilst this is certainly an important question in need of proper consideration, it is not necessary (nor feasible) to provide a full account within this paper. All that is necessary at this point is to note (a) the positive relationship between foresight and freedom as a choosing subject—simply, that more foresight is better, and (b) that AM presents significant risks to subordinate foresight.

The subsequent sections explore exactly how AM undermines the foresight of subordinates and thus perpetuates the foresight endangerment problem. First, I discuss the technical face of this problem, and how it is exacerbated by efforts to make algorithms more resilient. I then go on to explore the social face and explore reasons why the transfer of power from a social to a non-social agent weakens the epistemic position of the subordinate.

## 3 The technical face of algorithmic foresight endangerment

To fully develop the technical face of the foresight endangerment problem, I start with reviewing the technical aspects of algorithmic management, underscoring how unpredictable outcomes is an inherent feature of its operational processes. I then consider how this unpredictably registers in the experiences of workers managed by algorithms, focusing specifically on digital platforms like Uber and Lyft. A clear connection is drawn between algorithmic unpredictability and diminished worker foresight and, ultimately, the endangerment of their freedom as a choosing subject. I conclude the section by reflecting on the limits of ‘explainable AI’ in addressing the technical face of the foresight endangerment problem.

### 3.1 Algorithms: unexplainable and unpredictable black boxes

Algorithms are not a new technology. We have long used them as a systematic method for solving problems and performing complicated calculations. What has changed in recent years, however, is their sophistication and calculative potential due to major advancements in computing power, data availability in the digital age, and key innovations in mathematical theory. These areas of progress have enabled the programming of sophisticated algorithms based on machine learning (ML). Deep learning algorithms, which require massive amounts of data and computational resources, have only become feasible only in the last two decades due to recently developed hardware like graphics processing units (GPUs) and tensor processing units (TPUs).

The vast potential of algorithms due to these technological advances brings forward incredible opportunities, but also serious ethical concerns given our limited ability to understand how they function. Traditional algorithms follow an order of operations based on explicit, human-readable rules. This means that independent observers can trace how these algorithms reach their automated decisions. A classic example would be a basic software used for preparing one’s taxes. If there is confusion about how the software concluded the amount owed, one can simply reverse the steps the software took in calculating that amount. Or to put it another way, if one sufficiently knew the tax software’s programmed rules, they could perfectly predict what the algorithm would output based on the inputs.

Machine learning algorithmic systems, on the other hand, develop their own decision-making logic that emerges from data-driven training. After exposure to reams of data, these algorithms detect patterns that are too complex or subtle for humans to notice and make determinations based on observations of those patterns. The technical process is staggeringly complex. Modern ML models, like deep neural networks, contain millions, sometimes billions, of parameters and process data through multiple layers of abstraction resulting in highly intricate representations. The overwhelming number of parameters plus the way they interact across layers ultimately makes it extremely difficult to trace how models generate their outputs (or, in the case of AM systems, make decisions). Even developers of these systems often do not fully understand how these models are generating their outputs.

Advanced (machine learning) algorithms have thus been described as ‘black boxes’ (Gryz and Rojszczak, 2021). This expression, which has been used in various fields and contexts, refers to a system or device whose internal workings are not visible or understandable to an outside observer. The black box phenomenon has been widely discussed and associated with all kinds of ethical concerns (Von Eschenbach, 2021; Ajunwa, 2020). Our purpose here is to underscore its relation to the *unpredictability* of algorithmic outputs. Where the outputs of traditional algorithms can be predicted based on a sound knowledge of the rules they are programmed to follow, the decisions of advanced (machine learning) algorithms are not predictable in the same way and often appear random to users or subordinates.

This poses an obvious problem for those under the power of an algorithm. To be managed by an unpredictable decision-maker creates an environment characterised by diminished foresight into the consequences of one’s actions. Simply, if one cannot know what kind of reactions their choices will generate in a manager—i.e., the determinations of their *algorithmic manager*—then they have less capacity to make choices that will lead to their preferred ends. As we will see through the examples below, the technical causes of algorithmic unpredictability render AM a form of managerial oversight that harms the choice navigability of subordinates.

### 3.2 The technical face worsens: the resilience-predictability paradox

Before visualising this problem as it exists in the digital platform economy, it is worth first underscoring a key paradox inherent to the technical face of the foresight endangerment problem: as algorithms become more useful, they equally become more unpredictable. Hosanagar (2020, p. 106) has dubbed this phenomenon the ‘predictability-resilience paradox’, which he describes as follows:

Today, some of the most accurate machine learning models that computer scientists can build are also the most opaque. As machines become more intelligent and dynamic they also become more unpredictable. This suggests a fundamental conundrum in algorithm design.

As the paradox discloses, a priority must be established when designing algorithms. We can either create systems that are highly predictable but unable to adapt when confronting “problems they were not prepared for and therefore cannot solve.” Or we can train them on “messy real-world data to create resilient but also unpredictable algorithms.” Hosanagar cites the famous case of Google’s Go-playing computer programme AlphaGo as exemplary of this tension. AlphaGo used two types of machine learning to develop its own play style. First, it underwent a process of supervised learning, where the algorithm was trained on data from millions of plays made by Go champions in previous games. Second, AlphaGo improved its skills through reinforcement learning by playing millions of games against itself and refining its strategy based on the outcomes of those matches.

AlphaGo revealed its Go-playing prowess in 2016, defeating professional player Lee Sodel in 4 of the 5 games known as the DeepMind Challenge Match. Beyond the notable success of the algorithm, what is of particular interest is how AlphaGo won. On move 37, AlphaGo made a play that completely baffled observers, including Sodel who thought it was a mistake. Later reflections would reveal its brilliance. European Go champion Fan Hui, for instance, called it ‘So beautiful. So beautiful’ but also noted that, “It’s not a human move. I’ve never seen a human play this move” (Hosanagar, 2020, p. 104). The storey of AlphaGo gest even more interesting. Google developed a second iteration of the programme called AlphaGo Zero. This time, the algorithm was not trained on any human data (i.e., moves made by previous Go players) through supervised learning. Instead, it started with no prior knowledge of the game other than the basic rules and learned its playstyle from playing countless games against itself via reinforcement learning. The result? AlphaGo Zero beat the original AlphaGo 100–0 when they faced off in a tournament.

Again, the success of the (new and improved) algorithm is impressive. But for our purposes, the critical point is the heightened unpredictability of AlphaGo Zero. As Hosanagar notes, ‘Many commentators used the word ‘alien’ to describe the moves made by AlphaGo Zero” (p. 122). The original AlphaGo was highly resilient and could adapt to one of the best Go players in the world, but with moves that were highly unpredictable to the audience. Then came AlphaGo Zero, an algorithm that we even more resilient in adapting to the moves of its predecessor, but equally more unpredictable in its play style.

This trade-off between resilience and predictability has significant real-world implications, particularly in the platform economy, where algorithms already play a central role in managing workers and provisioning services. Uber and Lyft, for example, use machine learning algorithms to determine driver pay, facilitate surge pricing, and make ride assignments (Uber Technologies Inc., 2018). As with AlphaGo, these algorithms are highly effective at optimising operations and highly resilient to new situations that arise. However, their determinations can be experienced as unpredictable, which has serious consequences for drivers. These unpredictable and *unforeseen* outcomes can make it difficult for drivers to know how best to organise their work so as to promote their chosen ends. Herein lies the link between the technical obscurity of algorithmic management systems and the diminishment of foresight into the consequences of one’s choices.^2^

### 3.3 The experience of the technical face: managerial randomness

The social consequences of management by unpredictable algorithmic systems—including a loss of choice foresight—are starkly evident in the experiences of platform labourers. Researchers continually observe that workers managed by algorithms are often confused by managerial decisions (Möhlmann et al., 2023). Indeed, many are apt to describe disciplinary actions as seeming entirely ‘random’. Worker exasperation over inexplicable managerial activity was a common theme several in-depth interviews I conducted with independent contractors in the UK courier industry and their trade union representatives (Donoghue, 2023). Participants frequently made statements like: ‘X was deactivated for no reason’ or ‘(x) directive made no sense’ or ‘I was denied jobs and I do not know why’.^3^ Algorithmic managers evidently cultivate an environment tinged by notable unpredictability, a phenomenon that I will refer to as ‘managerial randomness’.

There are many examples that underscore the extensiveness of managerial randomness and its harmful consequences for workers and their freedom as choosing subjects. But first, a quick note of clarification regarding terminology. To describe the decisions of algorithmic management systems as ‘random’ is not a common framing in the literature. Instead, algorithms are usually described as opaque, unaccountable, or arbitrary. This is likely because algorithms are designed to be deterministic or probabilistic rather than random. I obviously, therefore, do not use the word ‘random’ here to suggest that the algorithm itself generates entirely random outputs but to underscore that there are contexts where algorithmic decisions are phenomenologically received as random to subordinates—a result of several factors including their complexity, opacity, and unpredictability (McGee and Hancock, 2024).

One of the most widely discussed instances of managerial randomness, in part because of its severe impact, is the reported experience of workers being ‘randomly terminated or deactivated’. As one Amazon Flex driver I interviewed explained:

So, it can be pretty hit-and-miss and there is quite a bit of pressure on us to keep good as such. But at the same time if with a lot of it is the case of if you do it right, then you’ll be fine. Then a lot of them people you’ll still do it right and no matter what people just do things that make it seem like you are in the wrong and *then people just get deactivated for no apparent reason* (Donoghue, 2023).

Evidence of managerial randomness is widespread. Those who monitor the social impacts of algorithmic technologies have noted that “There’s no scarcity of storeys about gig workers being underpaid, sometimes far below minimum wage. Or working inhumane hours. Or having their accounts (i.e., their livelihoods) *randomly deactivated*’ (emphasis added) (Zawacki, 2023). Nicole Moore, President of Rideshare Drivers United, explained to the Los Angeles Times that “they are not just ‘deactivating’ accounts here—they are firing workers, often *seemingly at random*, and stripping men and women of their livelihoods” (Cathcart, 2023). The Canadian union Gig Workers United has indicated that “deactivations have been happening at *random* to multiple couriers without any advanced notice” (Shaid, 2024). The Pay Up Protections (Ordinance 126,144) passed by the Seattle City Council in December 2022 have been described as “protecting against *random* deactivation” (Groover, 2024). Indeed, growing numbers of gig workers are organising around the objective of challenging platform companies’ ability to ‘randomly’ deactivate workers at will (Varghese, 2023).

Managerial randomness is not only experienced in relation to termination, but other kinds of employment-related decisions as well. Another contentious point that has led to significant outcry and worker mobilisation is how pay is calculated by algorithms. Again, we turn to Uber as an exemplary case. Uber’s computation of driver pay-outs can often appear random to drivers because of several interconnected functions of the algorithm. A prominent example is the use of ‘dynamic pricing’ to adjust fares in real time based on demand and supply. This practise has drawn the ire of drivers who perceive their earnings as inconsistent and incomprehensible because of the complexity of the surge multipliers. A major trade union of private hire drivers in the UK has been campaigning with:

“Another key demand [to] an end to Uber’s ‘dynamic pricing’ system… Drivers say the system is unpredictable and lacks transparency, making it difficult to plan earnings. Instead, the ADCU is pushing for fixed pay rates of £2.50 per mile plus 50p per minute, ensuring a more stable and sustainable income for workers” (Richardson, 2025).

There are other factors that that render pay-outs confusing for drivers like the use of upfront pricing, complex fare structures, and frequent updates to the algorithm’s overall pricing model. All of these factors in combination make it extremely difficult for drivers to anticipate or fully understand how their choices and eventual earnings are connected.

### 3.4 Managerial randomness and the loss of free choice

Managerial randomness directly illustrates how subjection to the power of an algorithm endangers individual choice foresight. Workers managed my algorithms labour under uncertain conditions due to the inherent unpredictability of these systems. This means that they are embedded within a choice architecture that is difficult to navigate.^4^ They confront the constant reality that choice X, which should result in reasonable outcome Y, could result in unknown outcome Z because they cannot be sure how the algorithm will process their data (and choices). It was noted earlier that choice foresight requires a causal and sequential environment, which we can now see is directly endangered by AM.

We can see the impact of this dynamic both within and outside of the workplace. Whilst in work, private hire drivers are forced to make choices they would prefer not to, under the compulsion to be overly conservative towards the unpredictable algorithm. The most worrying example is workers continuing to work even when feeling unsafe. Researchers have documented that drivers will not refuse a perceived dangerous passenger because of the risk of deactivation (Lefcoe et al., 2024). To be clear, drivers do have a contractual ‘right to refuse’ and Uber policy states that they will not be penalised for cancelling a trip due to safety concerns. Yet, if the desired end of a worker is continued employment, they ostensibly cannot choose to exercise that right because the consequences are unknown and include unfair deactivation. Indeed, it has been widely documented that Uber drivers operate in a ‘climate of fear’ regarding the omnipresent possibility of being unexpectedly deactivated.^5^

The prospect of random deactivation has consequences for workers outside of the workplace, too. A 2023 survey by the Asian Law Caucus (2025) and Rideshare Drivers United of 810 Uber and Lyft drivers found not only that two of three drivers have experienced temporary or permanent deactivation, but that “81% of [respondents] said driving on Uber and Lyft apps was their primary source of income.” Thus, it is unsurprising that following these deactivations, many are sent “into unexpected financial crisis” and “struggled to make ends meet, including 18% who lost their car and 12% who lost their homes after deactivation.” Uncertainty regarding income, we saw, is also related to how the algorithm calculates payments. Unpredictable wages, as the ADCU notes, make earnings-related planning difficult for workers. The union is pushing for a flat rate, not one that is dynamically calculated, so that workers can make work-related decisions with knowledge of what will result from their choices.

Working in uncertain conditions, due to random managerial outcomes, is a direct threat to the subordinate’s liberty to exercise of rational agency—i.e., the ability to organise our life according to self-chosen ends.^6^ Random fluctuations in one’s income—including the potential to lose one’s job seemingly out of nowhere—poses serious complications for making key life choices, or at least sustaining the conditions that allow one’s life choices to flourish. There are many actions individuals may take or paths they might pursue as expressions of their personality that require future-oriented thinking. A person who wants to have children or buy a home for their ageing parents, for example, might first consider the financial viability of such courses of action over the long run. Such calculations are an integral part of being a free choosing subject and living a self-defined life, as they are the foundation of pursuing a rational life plan that amounts to the realisation of one’s personality.

But those kinds of calculations require that one have reasonable foresight about his or her future. This includes the consequences of one’s present choices, in particular those choices that can impact his or her livelihood and income. Yet, the person under the power of an algorithm, such as the digital platform worker we have been discussing, is denied such foresight because their livelihood and income could be unpredictably severed at a moment’s notice. Surely, every worker is perennially exposed to some risk of losing their job. But the unpredictability of such an occurrence can vary wildly from one employment relationship to another. The algorithmic employment relationship—i.e., algorithmic management plus the independent contractor status—leads to widespread experiences of randomly being fired with zero foresight nor explanation. This deprives subordinates of algorithms the capacity to reasonably foresee the very serious consequences of their choices, and thereby strip away their capacity to live freely as a fully choosing subject.

### 3.5 What about explainable AI (XAI)?

An important question worth considering is whether recent developments in explainable AI (XAI) offer any solutions to the problem of foresight endangerment by AM. The XAI field is developing various techniques to help render algorithmic outputs more intelligible, including counterfactual explanations, actionable recourse, and human-centred design. Proponents allege that these techniques restore a degree of agency to those impacted by the decisions of algorithmic and automated systems. For example, a rideshare driver entitled to counterfactual explanations might (theoretically) receive a notification saying: “If you had accepted 3 more trips during Friday’s peak period, you would have qualified for a £75 bonus.” Or they could (theoretically) benefit from actionable recourse, with recommendations for concrete changes they could make to improve their earnings, like: “To be eligible next week, try accepting 90% of trips between 4 and 7 pm.” In both cases, important information is conveyed to the worker about how they could (have) improve(d) navigated to their preferred end of generating more income.

The efforts behind XAI include further attempts to make explanations intelligible to algorithmic subjects through human-centred explainable AI. HCAI aims to improves subordinates’ comprehension of algorithmic systems’ decisions by offering explanations tailored to their specific needs, contexts, and even cognitive capacities. Presenting information in accessible formats—i.e., visual summaries, accessible language, or simple metrics, and so on—can enhance their ability to understand how decisions are made about them and, ultimately, support more informed interaction with automated or AM systems. In short, HCAI strikes at the long-noted problem that many, if not most, individuals simply do not have the training or expertise to make sense of technical explanations regarding how an algorithm reached a particular decision.

All these areas of progress are undoubtedly important, and many researchers have noted their necessity for subordinates of algorithmic managers (Nagaraj Rao et al., 2025). XAI techniques do clarify and improve the interpretability of algorithmic outcomes which can lead to the correction of erroneous (and unjust) outcomes—when they can be effectively implemented.^7^ That capacity, whilst valuable in itself, confers other potential benefits like illuminating and mitigating biases in algorithms that that unfairly distribute rewards and punishments. With all this in mind, however, it would be premature, if not incorrect, to conclude that XAI currently offers a serious or complete solution to the (technical face of the) foresight endangerment problem as we have begun to develop herein.

The problem is that whilst XAI can improve retrospective understanding, it does not resolve the core issue at the heart of foresight endangerment: namely, the inability of subordinates to reliably predict the (algorithmic) consequences of their choices *at the moment those choices are made*. This is because XAI techniques (in most contexts) explain algorithmic decisions *post-hoc*. That is, XAI does not generally provide predictive insight into what will result from a given choice before it is made (ante-hoc); rather, it explains why a decision was made after the fact (post-hoc). Liao and Varshney (2021) explain how:

At a high level, XAI techniques fall into two camps: (1) choosing a directly interpretable model, such as simpler models like decision trees, rule-based models, and linear regression; (2) choosing a complex, opaque model (sometimes referred to as “black-box model”), such as deep neural networks and large tree ensembles, and then using a post-hoc technique to generate explanations.

This dynamic is generally referred to as the “performance-interpretability tradeoff,” underscoring that algorithmic design is beset—much like the predictability-resilience paradox—by a difficult tension regarding the possibility and ease of explaining outcomes. For a labour platform like Uber or Lyft, option (1) is not desirable nor possible, as the service provided by these companies requires sophisticated machine-learning algorithms. This means that the only kind of explanation that can be provided by these companies is post-hoc, but this, as we noted, is principally unresponsive to the core issue of foresight endangerment. If a worker makes a choice believing it entirely appropriate to do so, yet is subsequently deactivated because of it, learning later why the deactivation happened is much less helpful than having known it would happen ahead of time.

Beyond their unresponsive to the core issue, post-hoc explanations are also severely limited for enhancing subordinate foresight in another important way. AM systems—especially those used by digital labour platforms—are dynamic, adaptive, and continually augmented. This means that even when explanations are available after the fact, the rules by which the system operates may have already changed, rendering the knowledge obtained from the prior explanation obsolete. In the workplace, for instance, a worker might be told how prior decision *Z* led to some outcome *Y*, but they cannot be certain how a similar choice *~Z* will be assessed by the algorithm in the future.

It must be stressed that the unpredictability of algorithms, and the inability to provide clear ante-hoc explanations of what will happen in response to choices, is not simply a result of poor explanation or biassed design. It is an unavoidable feature of algorithmic optimization. The causal link between a worker’s action and its outcome is often obscured not only by opacity, but by the algorithm’s fundamental logics. Sophisticated algorithmic management systems, like Uber’s platform, are inherently difficult to predict because they use adaptive, data-driven models that continuously update in response to real-time conditions, making their outputs non-deterministic. These systems attune to multiple shifting objectives—such as efficiency, demand balancing, and anti-gaming—so the same actions (i.e., worker decisions) can produce different outcomes depending on broader contextual factors. These conditions explicitly undermine a subordinate’s ability to act with strategic foresight, even if they are provided with technically sound post-hoc explanations.^8^

The concept of foresight endangerment thus refers to a distinctive epistemic harm that is not addressed by existing XAI techniques: the structural unpredictability of outcomes within algorithmic systems. Whilst XAI may empower users to understand past outcomes or identify potential avenues for appeal, it does not reliably restore the conditions needed for full rational agency— namely, a stable and intelligible connection between action and consequence.

## 4 The social face of algorithmic foresight endangerment

This section turns to the social face of the foresight endangerment problem. This face emphasises what is lost by transferring managerial power away from a human to an algorithm. I frame this transition as a process of disembedding the exercise of power from a social to an a-social relationship, the former being constituted of two social agents and the latter of software and a user or data subject (Moore, 2024). There are major implications of the disembedding process for the foresight of subordinated persons.

First, it forecloses their access to streams of information they could draw upon to better understand and predict managerial decisions. Humans, as social beings, have an intimate awareness of and capacity to interpret countless signs and cues communicated through social interaction. We develop this ability through the process of socialisation, something entirely beyond the reach of algorithms who are instead trained on data. When managers participate in this medium of exchange, as human managers do, subordinates have unique tools at their disposal to understand and predict managerial activity.

Second, subordinates of human managers are empowered by *social processes* to seek restitution and correction from poor managerial decisions. The problem of holding algorithms accountable has been discussed extensively. Arguably the most suggested solution is a ‘right to explanation’ (Vredenburgh, 2022). Notice that this, which many point to as an indispensable part of ethical management, is something that human managers are easily capable of providing. On top of that, the processes that exist for challenging poor managerial decisions in the world of work, such as collective bargaining or employment tribunals, are fundamentally social in nature. The embedding of managerial procedures in a social relationship, as opposed to an algorithmic one, greatly enhances the predictability of outcomes by offering routes to contest erroneous arbitrary decisions.

### 4.1 The human manager: the advantages of a social employment relationship?

In standard employment, the actions and behaviour of subordinates (or workers) are overseen and directed by a human manager.^9^ Managerial activity and decision-making, therefore, unfolds on the familiar terrain of social interaction, as both parties are directly interacting with a social agent.^10^ It is the embedding of managerial activity within a social relationship, and its mediation through social interaction, that is critically important for the choice freedom of subordinates.

Social interaction is a deeply complicated and intricate process of individuals engaging with one another through the exchange of information, ideas, emotions, and behaviours. What seems quite basic and commonplace to us is miraculously made possible by the interplay of numerous different and complex capabilities. Interpreting and responding to each other’s actions, intentions, emotions, etc., to foster mutual understanding and cooperation requires core human skills: including but not limited to simulating the perspective of others, social cognition, verbal and non-verbal communication, contextual understanding, emotional intelligence, among other critical abilities. The interaction of these abilities and processes allows individuals to navigate social environments effectively by deciphering the meaning behind others’ actions and then responding appropriately.

AM dissolves the centrality of these dynamics and abilities as managerial activity is no longer embedded in a social exchange between two social agents. Instead, the manager-subordinate relationship is facilitated through non-social interaction between ‘software’ and a ‘data subject’. In what follows, I explore the potential risks to the choice freedom of subordinates when managerial power is disembedded. I focus on three specific aspects of social interaction and consider what their dissolution means for the freedom of subordinates as choosing subjects. I concentrate on these aspects in particular because their presence (or absence) reveals the critical differences between human and algorithmic management, especially from the point of view of the subordinated. Subsequently, we are able to see more clearly the possible threats to subordinate freedom posed by non-social management via AM.

#### 4.1.1 Knowledge of social scripts and contextual awareness

The first aspect of social interaction that ought to be emphasised is its governance by *social scripts* that people come to know through constant socialisation. The symbolic interactionist school of sociology has importantly revealed the significance of these subtle and often hidden protocols in social life. These researchers have theoretically and empirically illuminated how our social exchanges are structured by a constant ‘interpretive process… [of] self-reflective individuals symbolically interacting with one another’ (Denzin, 2004, p 82). The result, they argue, is that “all social action consists of social practises, situated in time–space, and organised in a skilled and knowledgeable fashion by human agents” (Giddens, 1981, p. 19). In this sense, social interaction includes individuals making constant micro-judgements about the present moment based on a history of socialisation within and experiences of the ‘circuit of culture’ in which they are embedded (Du Gay et al., 2013).

Take just one example: upon learning that a worker has suffered a loss in their immediate family, other members of a firm (managers and workers) will likely be moved to take extraordinary steps to lighten that person’s workload. This ‘social practise’ of covering a grieving person’s workload is a ‘joint act’, and it is from the composition of such joint acts that emerges the ‘social life of a human society’ (Blumer, 1981, pp. 136–169). Workers and managers, as subjects equally embedded in a shared ‘circuit of culture’, are disposed to interpret social events with similar understandings and meanings—they will both likely feel a social compulsion to act in support of the grieving person.^11^ It is on these grounds that Manford Kuhn, a pioneer of symbolic interactionism, defined human behavioural interaction as “purposive, socially constructed, coordinated social acts informed by preceding events in the context of projected acts that occur” (Carter and Fuller, 2015).

Under an algorithmic manager, managerial decisions are no longer mediated by the social scripts that define the culture in which the subordinate is embedded. Algorithms, unlike humans, have zero exposure or awareness of social contexts and therefore do not interpret events from the social scripts so engrained in the human ways of being-in-the-world (Heidegger, 1962). They are not raised and socialised in a community wherein they absorb all the subtleties encoded in millions of social interactions, like social cues, norms, and mores. The subordinate of an algorithmic manager thus suffers a reduced capacity to predict how algorithms may react to (new) circumstances and events, including their choices at work.

The testimony of a courier’s experience working with Amazon Flex elucidated this problematic dynamic. This participant described how they were sent into central London for their first ever shift, which proved highly difficult. As the hour grew late, they decided to return a dozen or so parcels to the depot instead of knocking on people’s doors inappropriately late in the evening. The next day they received an ostensibly automated email saying: “We fully expect you to weather the circumstances and try and deliver every parcel.” Following receipt of that email from Amazon, the participant:

…email[ed] them back saying my side of things, like this is the first time I’ve ever done it, I wasn’t particularly sure of the app, you made me go to Central London in you know four hours’ time. And I just said, you know, “as a first time surely you can cut me some slack.” Then the email I got back was pretty much like, “we do not really care about that. Whatever the circumstances, we expect to you deliver every parcel” (Donoghue, 2023, 115).

This courier was clearly counting on a system of management capable of interpreting the circumstances surrounding their choice not to continue deliveries through the lens of a shared circuit of culture. The supposition that one is entitled to ‘some slack on their first day’ is a widely expected norm based on our historical experiences entering new social spaces and institutions—both extending to and receiving this courtesy from others. It is a practise that is rarely stated anywhere in explicit terms but known to all because, to quote Kuhn, it is a “socially constructed, coordinated social act” of which we are “informed by preceding events” and experiences. This courier did not magically whip up this expectation; they anticipated it as a staple feature in the script of their cultural circuit.

Other contextual social knowledge is relevant in such a situation. A courier in this position, as the participant confirmed, might begin to suspect that it would be inappropriate to make a delivery after a certain time of night. So, they confront a choice: whether to continue ringing doorbells after a socially unacceptable time or return to the depot with undelivered parcels? Part of making this choice will inevitably involve attempting to surmise the consequences of failing to complete all deliveries, which in turn requires predicting how their manager will react. Observance of social norms is a key part of their personality, and they want to make a choice that aligns with their propositional attitudes. They may feel free to make that choice and express their personality because they can predict what will happen if they do.

The worker overseen by a human manager is thus equipped with the foreknowledge that their supervisor will, at a minimum, understand the reasoning behind their decision and incorporate that reasoning into their response. No such equivalent opportunity exists with an algorithmic manager. Not only is the algorithm completely blind to the world of social norms, but it is also inherently incapable of contextualising explanations given by workers to justify work-related decisions. In other words, in this moment of calculation, the courier simply has no idea how the algorithm will appraise their decision. It may be the cause of some subsequent sanction down the line, but they have no way of knowing. This lack of foresight hinders their ability to approach this decision as freely choosing subject as they cannot weigh the reasonably likely consequences.

#### 4.1.2 Empathy and simulation

A second distinct feature of human socialisation is our tendency to construct theories of mind that help us intuit the mental states (i.e., beliefs, desires, intentions, and emotions) of others. The construction of such theories allows individuals to take the perspective of others, predict their behaviour, and understand their way of thinking. Various disciplines have identified or theorised different ways we achieve this. One prominent explanation from the psychological tradition is simulation theory, which suggests that we can use our own mental states as a model to infer what others are thinking or feeling (Legerstee et al., 2013). In short, we mentally simulate their disposition by recalling our experiences in similar conditions. Some neuroscientists have argued that this process is supported by neural mechanisms like mirror neurons (Brooks, 2011). The ability to put ourselves in another’s shoes, according to this theory, critically enables our capacity to predict others’ behaviours and respond appropriately in social interactions.

The sociality of the standard employment relationship—as opposed to the algorithmic employment relationship—confers a unique power upon subordinates to read and interpret the subtle signals that disclose the mental states of a superior. Access to such information empowers subordinates to make choices with crucial insights derived from simulating potential managerial reactions to their actions. This enhanced capacity of foresight into the managerial response of one’s choices results in a more navigable choice architecture. Put another way, subordinates of a human manager are embedded in a social relation that where they can simulate scenarios to inform their choice-making to more easily realise their goals.

It is also worth considering the potential benefits of simulation going from the other direction. Human managers are capable of simulating the relevant mental states of both subordinates, and those they interact with. A human manager, for instance, can empathise with the above dilemma confronted by the worker and understand (from related personal experiences) the discomfort of disturbing people late at night. Additionally, the manager can equally empathise with a customer who would find it quite irksome to have their doorbell rung very late at night. Both of these sources of experiential data, plus knowledge of social norms, creates a more holistic picture by which the manager will judge the worker’s decision to return with undelivered parcels.

The courier can reasonably expect that from this holistic picture, which includes empathy generated considerations, a human manager will have a complete understanding of the courier’s decision-making process and respond accordingly. Or at least, for a newly employed courier the boss may demonstrate lenience out of respect for the courier’s likely discomfort at violating social customs and norms—and then inform them what to do in the future. Algorithms, obviously, lack the critical capacity of mental simulation and are completely ignorant of the rich and socially significant data it creates. They cannot simulate what it is like to be a worker (or customer) in that situation. Subordinates of an algorithmic manager, then, cannot count on this kind of mental simulation data when making calculations about the consequences of their choices. The courier cannot expect the algorithm to empathise with the unique and precarious context of his situation. This further deprives him of the ability to foresee the consequences of choices, ultimately undermining his ability to choose freely at this critical junction in the production process.

#### 4.1.3 Information rich communication

When managerial power is diesmbedded from a social relationship to an asocial one, a whole stream of information that emerges from social communication itself is eliminated to the detriment of the subordinate and their choice foresight. Through continual interaction, the tendencies and dispositions—including negative or harmful ones—become known to subordinates. Furthermore, as workers and human managers operate in a ‘culture sharing group’, social information is spread amongst the members of that group, increasing the stock of knowledge about one another. In a culture sharing community, knowledge of *P’s* experiences allows *Y* to reasonably foresee manager *M*’s reaction to similar circumstances.

But we can go further in our analysis of what is lost beyond learning from repeated interaction. Indeed, it is possible for subordinates to detect key social information even without the exchange of words. Sociologists have long demonstrated that social information can be conveyed in the most subtle of cues, movements, intonations, and so on (Jones, 2017). Non-verbal communication is an integral part of social life, and it mutates and develops from one social context to another (Phutela, 2015). It is, therefore, not surprising that research into traditional workplace communication reveals that “both verbal and non-verbal communications at the workplace played big roles in ensuring the smooth flow of the company” (Yusof and Rahmat, 2020).

In the standard employment relationship with a human manager, a worker may start to collect hints that they are approaching a disciplinary response. Through subtle (but also not subtle) indications, the worker may detect that their performance is not up to par. In this sense, they may benefit from an awareness of their precarious situation and are able to take the necessary steps to find firmer ground. Algorithms do not and cannot engage in such delicate communication. Digital platform workers do receive performance statistics and have a general theory of what those statistics must look like to avoid deactivation, but this is a very brute indicator with the exact line often being unknown. Furthermore, workers managed by an algorithm may be at risk because of some unknown reason in the algorithm unrelated to obvious performance statistics—a possibility of which they have no awareness of nor could have awareness of.

These more delicate modes of communication that convey information about likely managerial behaviour cease to exist in the disembedded algorithmic managerial relationship. Algorithmic bosses deprive workers of insight into the ‘mind’ of the power that manages them. Workers cannot ‘get to know’ an algorithm through social interaction, and slowly gather information regarding how it may react to their choices or novel circumstances that may arise. There is no ‘working relationship’ with an algorithm based on past experiences and shared understandings that develop over time. Subordinates of an algorithm cannot ‘test or tease out’ a potential choice and see the response of the manager before actually making that choice. Instead, they confront an asocial power whose outcomes can appear entirely unpredictable and random. The dissolving of these pathways for information gathering for subordinates puts them in a much more difficult position to know the consequences of their decisions and thus ultimately navigate towards their preferred ends as a full choosing subject.

#### 4.1.4 What about biassed human managers?

Some will rightly question: are human managers not capable of ‘arbitrary’ and ‘random’ reactions or interferences that defy the norms and scripts of the shared circuit of culture? The answer is undoubtedly yes. However, we have seen that their crucial distinctions between a human manager capable of an arbitrary reaction and algorithmic systems. Most notably is the extent to which their respective arbitrariness or ‘randomness’ is predictable and navigable (and contestable as explored in the next section). A worker may very well find herself under the prerogative of a foul manager, which is likely to lead to unfair treatment. However, knowledge of this unfortunate disposition grants workers some ability to factor it into their calculations when making choices in the workplace. For instance, they may be likely to take a more conservative approach to exercising certain discretions based on the wariness of that manager.

To be sure, a worker’s capacity to evade partial or biassed treatment does not make it less morally problematic, but it highlights a key difference between the human and algorithmic managers. Workers are unable to detect the ‘character’ or ‘mood’ of algorithms to inform their choice calculations as they can with other humans. This means that workers managed by humans retain an improved ability to ‘predict’ what outcomes may result from their actions even whilst dealing with a difficult manager. For example, it may become apparent to *W* that manager *M* is ‘having a bad day’, and so they may conclude it is riskier to make some particular choice (asking for a raise/time off/etc.) today and wait for another more opportune moment.

### 4.2 Contesting anti-sociality and creating managerial causality

There is one further aspect of traditional social management that may have important implications for the freedom of subordinates: namely, the practical and existent possibilities for holding human anti-social behaviour accountable. If we return to the world of work, we see that there are legal, judicial, and cultural mechanisms that curb anti-social managerial activity.

Traditional workplaces defined by a social employment relationship are designed, albeit imperfectly and to considerably varying degrees, to procedurally constrain such dispositions through company handbooks, grievance procedures, HR departments, union representation, employment tribunals, and so on.^12^ These *social* processes and institutions exist to ensure that exercises of managerial power are properly targeted when used. Whilst a power imbalance necessarily persists in the standard employment relationship, sanctions (supposedly) cannot be exercised with pure impunity. Cheques and balances exist to eliminate or, at a minimum, challenge unjust outcomes, which provides some degree of security about the expected consequences of choices at work.

The capacity of workers in standard employment to contest anti-social managerial activity is no mere theoretical speculation. In the case study I conducted with standard employees in the UK courier industry verified that workers embedded in a traditional employment relationship have considerable recourse against what they experience or perceive to be abuses of power, including disciplinary action that has no justifiable basis. Participants cited the existence of many such institutions that provide such protection. A union presence was frequently noted as ensuring that ‘someone has your back’, especially “in terms of disputes, minor disputes. It happens on a weekly basis” (Donoghue, 2023, pp. 151–152). Indeed, employees documented how the union has been able to secure certain controls on disciplinary procedures in the collective bargaining agreement, such as prohibitions on using tracking data for targeted discipline or that a union rep must always be present during disciplinary actions.

The employment tribunal was similarly cited by standard employees I interviewed as a key mechanism for challenging anti-social sanctions. One participant (who was also a union rep), when probed on what would happen if someone was unfairly or arbitrarily terminated, responded, “That would be ridiculous, and we’d go to like an employment tribunal with that or something.” (*Ibid.*, p.152). Employees’ sense of job security is thus further enhanced by the knowledge that, where the union itself is ineffective, another external social institution like the tribunal can activated. I emphasise the tribunal as a *social institution* because its proceedings unfold through social interaction, including requiring witness statements or testimony from managers and employers to better understand the specifics of a case. It is through these procedures that the intentions and actions of managerial discipline are revealed and scrutinised. Human managers as social agents can vocalise the reasoning behind their actions in a discursively observable way. It is unclear how an algorithm could similarly provide testimony, as it is a non-social agent incapable of explaining its thought processes.

We also should not discount the role of social norms and expectations play in shaping managerial activity. A key aspect of our socialisation is the encoding of norms around fairness, reciprocity, and helping others, as these moral values function as the social glue for producing social cohesion (Durkheim, 1984). Our natural tendency towards the prosocial treatment of others is well documented. We have an evolved, natural disposition to act fairly towards others, thanks to our unique capacity for intersubjectivity and cooperation (Tomasello, 2016). Algorithms, on the other hand, are not constrained by social expectation and moral intuition. Algorithms do not confront the social judgement and guilt of appearing partial in their treatment of workers. Whereas, if a human manager fired A for failing to make five deliveries and then did not do the same to B, this impartiality would violate basic social norms of fairness—and the force of social pressure around that deviation may come bearing down on them.

The combination of (a) the inherent sociality of human management and (b) the existing institutions designed to minimise antisociality, produces a mode of discipline that is experientially causal (to a considerable extent). In other words, the embeddedness of standard employment in a social relation (and the contestability of anti-social managerial activity) fosters a workplace where subordinates tend to experience disciplinary actions as caused by something of which they are knowledgeable. This means that sanctions are, on balance, a sequentially causal event: action, choice, or event *A* resulted in sanction *B*. Compare that our description of algorithmically-managed workers who confront the constant reality that choice X, which should result in reasonable outcome Y, could result in unknown outcome Z because they cannot be sure how the algorithm will process their data (and choices).

To experience discipline exercised by a superior in a sequentially causal manner is critical for individual freedom as a choosing subject. It plays a crucial role in enabling subordinates to reasonably foresee the consequences of their choices, because they can act based on the (strong) certainty of the events (i.e., consequences) that will follow prior events (i.e., personal choices). If we are correct in associating human management with managerial causality, and algorithmic management with managerial randomness, then we have good reason to be concerned about the fate of subordinate freedom given the growth of the latter throughout the social world. These associations would suggest that the expansion of AM will also amount to the expansion of the foresight endangerment problem.

## 5 Conclusion

The paper concludes that algorithmic management poses a significant threat to individual freedom because it undermines the foresight condition, which we have established is a necessary component of the freely choosing subject. The technical face of this threat arises from the inherent and unavoidable tendency for the outputs of advanced algorithms to be unpredictable. Moreover, this unpredictability issue is further exacerbated by the resilience-predictability paradox which renders this risk to the freedom of subordinates all the more prevalent in real world situations. We visualised how the technical face of the foresight endangerment problem unfolds through an analysis of the experiences of digital platform workers. Algorithmically managed workplaces are beset by managerial randomness, where workers experience arbitrary decisions (e.g., sudden deactivation or inconsistent pay) that they cannot anticipate or understand. This unpredictability strips workers of the ability to make informed choices aligned with their goals, ultimately eroding their choice freedom.

The social face of the foresight endangerment problem points to the consequences of disembedding managerial power from social relationships (between humans) to asocial relationships (between humans and algorithms). Subordinates of human managers enjoy a vast number of tools to predict managerial thinking that arise from the intricate and complex processes of social interaction. Humans, unlike algorithms, are aware of and constrained by the social scripts and circuits of culture. Human managers and workers can simulate the perspectives of one another. In a social relationship, there is a continuous exchange of information rich communication. These dynamics critically engender enhanced choice foresight for subordinates, and it is lost when managerial power is disembedded. The disembedding process fundamentally undermines workers’ ability to navigate their choices and pursue their rational life plans.

This paper thus underscores the need for greater scrutiny of algorithmic management systems, particularly their impact on individual freedom. Further exploration of how these systems disembed power from social constraints, and the consequences of such disembedding, is urgently needed. How can we preserve the social and predictable aspects of human management to safeguard the freedom of subordinates to algorithmic managers? Perhaps it will be necessary to consider philosophical solutions like extensive proscription of algorithms in certain contexts and go beyond technical fixes like rights to explanations.

## Data Availability

Requests to access these datasets should be directed to r.donoghue@essex.ac.uk.

## Footnotes

^1^Hirschmann (2003) captures this sentiment well: “Implicit in these introductory questions is the more fundamental issue of what the term freedom means. This is a central bone of contention among liberty theorists; but most, if not all, conceptions of liberty have at their heart the ability of the self to make choices and act on them” (3).

^2^It is worth underscoring here a subtle but important distinction between transparency and unpredictability. Outcomes can be made transparent after the fact, but that does not solve the problem of the AM systems being experienced as unpredictable decision-makers. I explore this further in the below discussion of explainable AI.

^3^A fuller explanation can of the methodology behind those interviews can be found in the chapter three of cited thesis.

^4^Cass Sunstein helpfully draws out importance of ‘navigability’ as a requisite of freedom. See: Sunstein (2019).

^5^The campaign Gig Workers Rising (2025), in a petition to Uber and Lyft CEOs, explains that “Every day, Uber and Lyft drivers work under a constant fear of having their accounts deactivated… The constant threat of termination, in addition to limited opportunity for recourse, means drivers are constantly in a state of fear. Drivers’ stability at work is entirely out of their hands. Instead, their future is decided by the whims of passengers and the companies.”

^6^In this reference to rational agency, I borrow from Mark D. Friedman and his interpretation of Robert Nozick’s *Anarch, State, and Utopia*. See: Friedman (2003. p. 18).

^7^An illustrative example is banks using XAI tools like SHAP to detect false positives in fraud detection or credit risk assessments. SHAP identifies which input features most strongly contributed to a model’s prediction, enabling human reviewers to spot implausible or context-insensitive reasoning and make more informed decisions (Mahajan and Shukla, 2023).

^8^We could further add that, in addition to the nature of algorithmic processing, algorithmic managers (or platforms) have an incentive to deliberately obscure the inner logics of these systems to prevent manipulation by users/workers and protect intellectual property.

^9^AM systems are finding their way into traditional employment settings. I am thus simply using the expression ‘standard employment’ to as a term of reference for an employment relationship with a human manager.

^10^Whilst the concept of a social agent is not without controversy, here I take it to mean a human person whose identify and sense of self was formed through a socialisation process.

^11^Of course, not all individuals will react to an event in the same way. There will be managers or colleagues that demonstrate indifference. The dynamic nature of the social world inevitably implies divergence from social practises, but these distribution may be exceptions and in some cases predictable.

^12^Some argue that power is rendered non-arbitrary, and therefore non-dominating, when it is procedurally constrained. However, others will say that power must also track the interests of those subject to it or be controlled by those under it’s mandate. See for instance: Lovett (2010) and Arnold and Harris (2017).
